# Occupational respiratory diseases in the South African mining industry

**DOI:** 10.3402/gha.v6i0.19520

**Published:** 2013-01-24

**Authors:** Gill Nelson

**Affiliations:** Division of Epidemiology and Biostatistics, School of Public Health, Faculty of Health Sciences, University of the Witwatersrand, Johannesburg, South Africa

**Keywords:** gold, platinum, diamond, miners, silica, asbestos, silicosis, migrant labour, autopsy

## Abstract

**Background:**

Crystalline silica and asbestos are common minerals that occur throughout South Africa, exposure to either causes respiratory disease. Most studies on silicosis in South Africa have been cross-sectional and long-term trends have not been reported. Although much research has been conducted on the health effects of silica dust and asbestos fibre in the gold-mining and asbestos-mining sectors, little is known about their health effects in other mining sectors.

**Objective:**

The aims of this thesis were to describe silicosis trends in gold miners over three decades, and to explore the potential for diamond mine workers to develop asbestos-related diseases and platinum mine workers to develop silicosis.

**Methods:**

Mine workers for the three sub-studies were identified from a mine worker autopsy database at the National Institute for Occupational Health.

**Results:**

From 1975 to 2007, the proportions of white and black gold mine workers with silicosis increased from 18 to 22% and from 3 to 32% respectively. Cases of diamond and platinum mine workers with asbestos-related diseases and silicosis, respectively, were also identified.

**Conclusion:**

The trends in silicosis in gold miners at autopsy clearly demonstrate the failure of the gold mines to adequately control dust and prevent occupational respiratory disease. The two case series of diamond and platinum mine workers contribute to the evidence for the risk of asbestos-related diseases in diamond mine workers and silicosis in platinum mine workers, respectively. The absence of reliable environmental dust measurements and incomplete work history records impedes occupational health research in South Africa because it is difficult to identify and/or validate sources of dust exposure that may be associated with occupational respiratory disease.

South Africa is a mineral-rich country. Although the mining of these minerals generates wealth for the country, it also causes diseases in the mine workers who are exposed to harmful dust.

Pulmonary silicosis, the disease most commonly caused by exposure to crystalline silica dust, was described in South African gold miners in the early 1900s – not many years after gold-mining commenced. Most studies since then have been cross-sectional, with only one large cohort study being conducted on white miners; long-term trends have not been reported. Currently, South Africa has one of the highest rates of silicosis in the world.

Silica is a component of igneous rock that is found throughout South Africa. Another harmful mineral that occurs commonly in South Africa is asbestos. Although much research has been conducted on the health effects of silica dust and asbestos fibres in the major mining industries in which exposures to these minerals occur (viz. the gold-mining and asbestos-mining sectors), little is known about their health effects in sectors in which they are accidentally mined. Exposure to these two minerals was the focus of a recent PhD thesis, the themes of which are illustrated in Table 1 (1–4). The first theme addresses trends in silicosis in the South African gold-mining industry over a period of 33 years, and the second explores the potential for occupational respiratory disease in the under-researched platinum- and diamond-mining sectors. This paper is a synthesis of three publications that emanated from the thesis, and it highlights the major findings.

**Table 1:** Summary of major findings from the thesis related publications

	Silicosis in gold miners (1)	Oscillating migration (2)	Asbestos-related diseases in diamond miners (3)	Silicosis in platinum mine workers (4)	Integrating narrative
Trends in silicosis in South African gold miners	The proportion of black miners diagnosed with silicosis increased from 3 to 33% from 1975 to 2007; in white miners it increased from 18 to 22%.	Health care costs were externalised away from the mining companies as a direct result of oscillating migration; this resulted in epidemics of diseases such as silicosis, tuberculosis, and HIV.			Increasing trends are due to the migrant labour system, poor dust control, an inadequate occupational exposure limit, and an ageing workforce with increasing durations of employment.
The potential for respiratory disease in under-researched mining sectors			Diamond mine workers are at risk of developing asbestos-related diseases due to the composition of the rock.	Platinum mine workers are at risk of exposure to crystalline silica and developing silicosis.	The PATHAUT database provides an opportunity for disease surveillance in miners of all commodities, including those in which risks of ill health are considered to be minimal.

## The gold-mining sector

Until the 1990s, there were very few studies on the extent of silicosis in South African gold miners (Table 2) (5–15). The only long-term cohort study, conducted on white miners, provides a clear illustration of the progression of silicosis long after retirement. Not surprisingly, the prevalence of disease in black miners in earlier years (8), when exposure to dust was for relatively short periods, was much lower than in white miners. Three subsequent studies of ex-miners from Lesotho, Botswana, and the Eastern Cape reported proportions of silicosis of up to 36% (10, 11, 13). By 2001, many years after short-term contracts had been phased out, the proportion of black employed gold miners with silicosis was 14 times higher than that reported in 1984 (8, 14).

**Table 2:** Studies of silicosis in South African gold miners, 1978–2009

Authors	Study design	Study period	Study population	Study site	Sample size *N*	Mean/range of employment (years)	Diagnostic tool	Proportion with silicosis	Limitations
White gold miners									
Irwig and Rocks 1978 (5)	Cross-sectional	1968 to 1971	Employed white miners aged 45–54	All areas	1,973	>10	Chest X- rays	6.8	White miners only* †
Hnizdo and Sluis-Cremer 1993 (6)	Cohort	1968 to 1991	White ex-miners – living and dead	All areas	984	23.5	Chest X- rays	14.0	White miners only†
Murray and Hnizdo 2005 (7)	Cohort	1968 to 2003	Deceased white gold miners	All areas	1,476	23.5	Autopsy	51.6	White miners only
Black gold miners									
Cowie and van Schalkwyk 1987 (8)	Cross-sectional	1984	Employed black miners	Orange Free State, SA	132,765	Not stated	Chest X- rays	1.4	Black miners only; no ex-miners; denominator included new recruits* †
Murray et al. 1996 (9)	Cross-sectional trend analysis	1975 to 1991	Deceased black gold miners	All areas	16,454	4.4–6.9	Autopsy	9.3–12.8	Black miners only; 62% of men employed for <5 years 78% younger than 40*
Steen et al. 1997 (10)	Cross-sectional	1994	Living black ex-miners	Botswana	304	15.5	Chest X- rays	26.6–31.0	Black miners only; selection bias; no current miners* § †
Trapido et al. 1998a (11)	Cross-sectional	1996	Living black ex-miners	Eastern Cape, SA	238	12.2	Chest X- rays	22.0– 36.0	Black miners only; no current miners* § †
Meel 2002 (12)	Cross-sectional	1997 to 1999	Living black ex-miners – hospital patients	Eastern Cape, SA	300	Not stated	Chest X- rays	34.0	Black miners only; selection bias (hospital patients); no current miners* § †
Girdler-Brown et al. 2008 (13)	Cross-sectional	1999	Living black ex-miners	Lesotho	624	25.6	Chest X- rays	24.6	Black miners only; no current miners* § †
Churchyard et al. 2004 (14)	Cross-sectional	2000 to 2001	Employed black miners older than 37	North West province, SA	520	21.8	Chest X- rays	18.3– 19.9	Black miners only; no ex-miners; healthy worker effect* § †
Park et al. 2009 (15)	Cohort	1999 to 2000	Living black ex-miners	Lesotho	553	26.1	Chest X- rays	27.0	Black miners only; no current miners; short follow-up of 1 year§ †

* cross-sectional study design

§ small numbers.

† insensitive diagnostic tool.

Most studies have been cross-sectional and have used chest radiography (a relatively insensitive diagnostic tool), and have had small study populations. Studies in current miners underestimate the extent of silicosis because many develop the disease only after they have left the mining industry. This is due to the long latency of disease after dust exposure.

## The diamond-mining sector

**Fig. 1:**
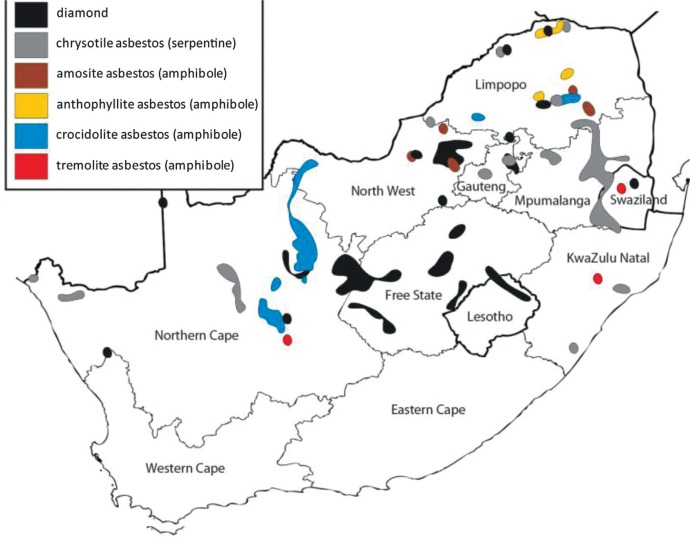
Map of South Africa showing geographical distribution of diamond and asbestos deposits [generated using ArcExplorer software (17)].

There is strong evidence to suggest that diamond mine workers in South Africa are at risk of asbestos exposure. Kimberlite often contains fragments of ultramafic rock that have a low silica and a high magnesium and iron content; these elements form the basis of amphibole minerals (16) that can occur as asbestos. Diamond deposits are also found close to asbestos deposits in several provinces (Fig. 1). Two reports, published in 1995 and 2001, respectively, describe airborne asbestos fibres in different diamond mines (18). Crocidolite asbestos was identified around the perimeter of the kimberlite pipe of a diamond mine in the Asbestos Hills in the Northern Cape (PJ Jordaan, unpublished data) and, in 2009, a sample of chrysotile asbestos was sent from a diamond mine in Limpopo province to the National Institute of Occupational Health (NIOH) electron microscopy laboratory for fibre analysis.

## The platinum-mining sector

**Fig. 2:**
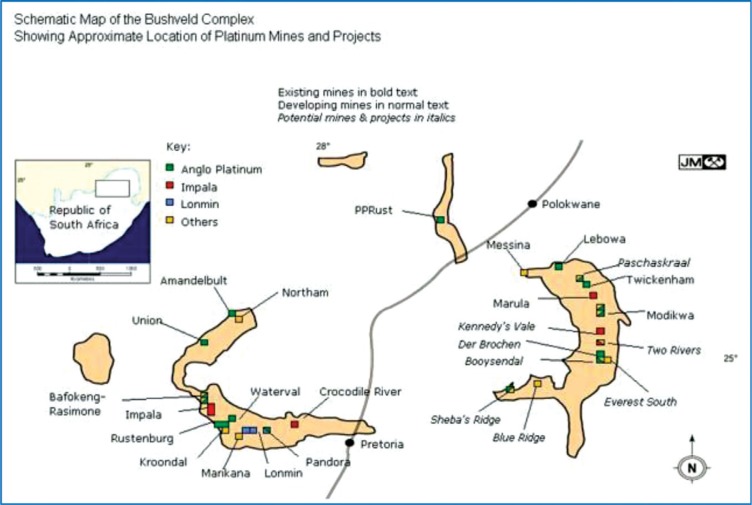
Map showing the platinum mines in the Bushveld Complex (19).

The platinum group metals (PGMs) are found in the Bushveld Complex in the northeastern part of South Africa (Fig. 2). As an igneous intrusion, the Bushveld Complex contains crystalline silica in addition to many other minerals and compounds. Miners of any of the minerals in the Bushveld Complex are therefore potentially at risk of exposure to silica dust.

There are very few available silica dust measurements from the platinum mines because the silica content of the dust is not routinely measured (20). One study calculated the crystalline silica content of crushed stope rock samples from two platinum mines as 0.45%, compared with 9.9 and 39.1% in two gold mines (21). The silica content of respirable dust samples was less than 0.2%, compared with 4.5% to 57% in the gold mines. A second study reported respirable silica dust concentrations ranging from 0.018 mg/m^3^ to 0.035 mg/m^3^ (22); this is much lower than the South African Occupational Exposure Limit (OEL) of 0.1 mg/m^3^ but higher than the American Conference of Governmental Industrial Hygienists (ACGIH) limit of 0.025 mg/m^3^. The only study of silicosis in platinum mine workers was a cross-sectional survey of 969 miners; silicosis was diagnosed in three, all of whom had a history of gold-mining (18).

The objectives of this PhD thesis were 1) to describe trends in silicosis diagnosed at autopsy in South African gold miners from 1975 to 2007; 2) to explore the possibility of asbestos exposure during the process of diamond-mining; and 3) to explore the possibility of developing silicosis during the process of platinum-mining.

## Methodology

### Data sources

There is no single database in South Africa from which comprehensive employment records and exposure data can be extracted. Therefore, a variety of data sources were explored to compile employment histories that were as complete as possible for each section of the thesis.

In terms of the Occupational Diseases in Mines and Works (ODMW) Act, the pathology division of South Africa's National Institute for Occupational Health provides an autopsy service for deceased miners and ex-miners for the diagnosis of compensable disease, regardless of the clinical cause of death or their disease status, provided the next of kin gives consent. Although autopsies have been performed for many decades, until 1975, all reports were paper-based. Details of each autopsied case are recorded in an electronic database that is part of the PATHAUT (Pathology Automation) System. The database provides for the recording of not only disease data but additional information related to demography and exposure (employment in up to five different mining sectors together with the years employed). The PATHAUT database is an important resource for surveillance and research on miners and ex-miners of all commodities. It contains the only data on occupational lung disease in the South African mining industry diagnosed by standard pathological methods that are far more sensitive and accurate than chest radiography. It currently comprises more than 105,000 autopsy records of miners from all population groups, all mining sectors, and all regions of South Africa dating back 37 years to 1975. The PATHAUT database was the primary and common data source used for identifying mine workers with occupational respiratory disease in all three mining sectors (gold, diamond, and platinum).

More comprehensive mining employment histories are recorded by the Medical Bureau for Occupational Diseases (MBOD) in Johannesburg. Until 1994, when the amendments to the ODMW Act were promulgated, examinations of white miners were conducted at the MBOD itself in central Johannesburg or at one of the sub-bureaus located in the vicinity of the gold mines. All records were kept at the MBOD. Black miners, on the other hand, were examined at the mines but their data were not routinely submitted to the MBOD. Nowadays, all examinations are conducted at the mines; data are sent to the MBOD when an occupational disease is suspected. Records are kept for all miners who apply for compensation for an occupational respiratory disease and are linked to the PATHAUT database by a unique MBOD number.

Files of diamond miners with asbestos-related diseases and platinum miners with silicosis were reviewed, specifically for employment histories, to identify those who might have worked in a mining sector other than the one of primary interest. Evidence of potential exposure to asbestos and employment in the gold mines was important to exclude the possibility of miners having contracted the disease of interest in an industry or mining sector other than the diamond or platinum mines, respectively.

Until 2004, when the electronic Mine Workers’ Compensation (MWC) system was implemented, the MBOD was entirely paper-based. Since then, all new applications to the MBOD are also recorded electronically. The MWC system consolidates and summarises information on work histories from the autopsy database, the MBOD files, and other sources. The MWC reports were reviewed to corroborate work history information obtained from other sources for diamond and platinum mine workers.

The Employment Bureau of Africa (TEBA) Limited was established in 1902 to assist the gold-mining industry in hiring mine workers. TEBA still deals primarily, but not exclusively, with gold miners. Each miner is assigned a unique industry number that remains his personal number for life even if he is subsequently employed in another mining sector. This number is recorded in the PATHAUT database. The electronic TEBA service history records were reviewed for the diamond and platinum mine workers to confirm that they had not been employed in the asbestos- and gold-mining sector, respectively.

Mining companies archive personnel data (albeit for a limited period and often in paper-based files). Where available, records were reviewed for specific job descriptions and to identify gaps in employment histories. For the studies on diamond miners, tailings and soil samples were collected from three different mines.

### Study designs

Silicosis in gold miners at autopsy was described using a time trend analysis over a 33-year period. The studies to investigate diamond miners with asbestos-related diseases and platinum mine workers with silicosis were both descriptive case series.

### Selection of study populations

Data were extracted from the PATHAUT database for each of the three study populations. For gold miners, all autopsies were included from 1975 to 2007. For the case series of diamond mine workers, cases were identified from 1975 to 2008, and for the case series of platinum mine workers, cases were identified to 2009. All gold miners were included in the trend analysis, regardless of whether they had worked in another mining sector or not. Diamond and platinum mine workers, however, were included in the respective case series only if they had not worked in another mining sector. Only miners who started working before the age of 24 and those with gaps in their work histories of less than one year were included in order to minimize the chance of including mine workers who had been exposed to asbestos or silica outside of the asbestos- and platinum-mining industries, respectively. The additional inclusion criteria are described in detail elsewhere (1, 3, 4).

### Disease diagnosis

Over the years, diseases have been diagnosed and graded by severity by experienced pathologists at the NIOH according to standardised methods. For the purposes of these analyses, silicosis was defined as the presence of palpable silicotic nodules on macroscopic examination of the lungs, which was then confirmed on microscopic examination. Asbestosis was diagnosed microscopically by the presence of diffuse interstitial fibrosis and two or more associated asbestos bodies. Pleural plaques were defined as circumscribed areas of dense acellular collagenisation with a basket weave appearance involving the parietal, diaphragmatic, and/or visceral pleura and with a minimum diameter of 5 cm.

### Statistical analyses

The relevant data for each aspect of the thesis were extracted from the statistical analysis system (SAS) PATHAUT database and transferred into a Stata IC 10 database for analysis. In each case, a repeat validation of the data was performed for the variables of interest, and the Stata dataset was amended with any additional data (e.g. employment histories) identified from the supplementary sources. Trend analyses on silicosis in gold miners were stratified by race. Trends in the crude proportions of miners with silicosis by year were assessed by means of simple linear regression with weighting by the inverse of the variances of the single year proportions. Binary logistic regression modelling with silicosis (1=present, 0=absent) as a dichotomous outcome was used to estimate associations with the explanatory variables. The three explanatory variables were age at death, duration of employment, and year of autopsy. Direct age and duration of employment standardisation was used to compare proportions of black and white miners with silicosis. The diamond mine workers with asbestos-related diseases and the platinum mine workers with silicosis were described in terms of their demographic characteristics, pathological findings, and occupations.

### Ethical considerations

Consent for autopsy examination can be granted by the next of kin in terms of the Occupational Diseases in Mines and Works Act (ODMWA) of 1973 (23) and the Occupational Diseases in Mines and Works Amendment Act of 2002 (Act no. 60 of 2002). This PhD protocol was approved by the University of the Witwatersrand Committee for Research on Human Subjects (M050228). Permission was obtained from the mining companies for the collection of samples of tailings.

## Results

### Silicosis trends in gold miners

**Fig. 3:**
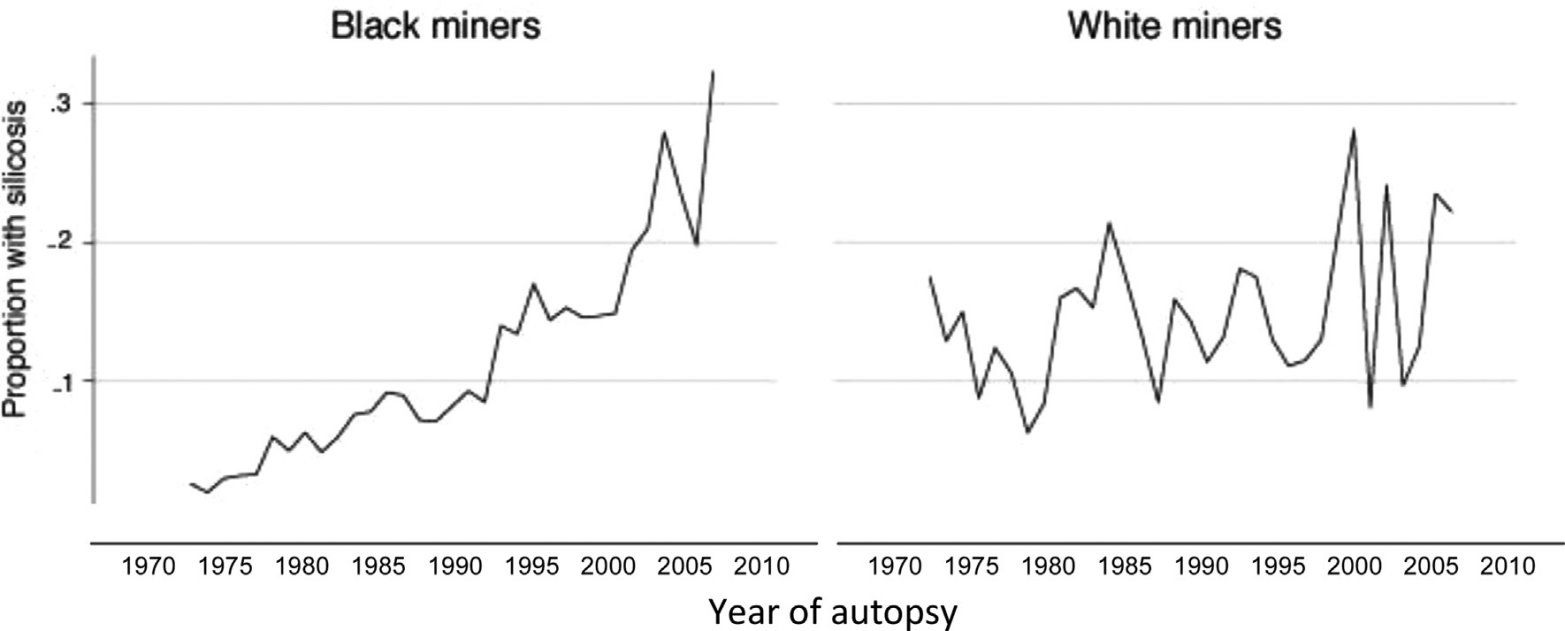
Crude population group-specific proportions of gold miners with silicosis, 1975–2007.

Of the 19,143 gold miners who died from external causes, 16,411 (85.7%) were black and 2,732 (14.3%) were white. The crude proportion of black miners with silicosis increased tenfold from 3 to 32%; the increase in white men was slight, from 18 to 22% (Fig. 3). More white miners had silicosis in 1975 (the proportion was six times higher than that of black miners) but, by 2007, this pattern was reversed (the proportion was 1.5 times higher in black miners). Overall, the proportion of black miners with silicosis was almost double that of white miners for the 33-year period.

Increasing age at death and duration of exposure explained much of the increase in silicosis.

The mean age at death of the black and white miners increased by more than 10 years over the study period. For the black miners, the mean age at death rose from 33.0 years in 1975 to 43.4 years in 2007, whereas the mean age at death for the white miners rose from 44.1 years in 1975 to 54.4 years. The mean duration of employment increased by less than 3 years for the white miners (from 17.5 to 20.1 years) but by almost 8 years, from 5.6 to 13.4 years, for the black miners.

The standardised proportion ratio (SPR) by race was 1.70 (95% CI 1.45–1.99); that is, the proportion of black miners with silicosis was 70% higher than that for white miners over the entire period, but this varied over the study period. When the analysis was repeated for miners dying from natural causes, the SPR increased to 2.27 (95% CI 2.12–2.43), most likely due to the association of tuberculosis with silicosis.

The overall age and employment duration-adjusted proportion of black miners with silicosis was higher than that of white miners for all years of autopsy other than 1975–1979 suggesting that a factor(s) other than increasing age and duration of employment was associated with the increase in disease. It is likely that black miners had higher intensities of exposure than white miners due to the dustier jobs in which they were (and still are) employed. This is supported by the fact that more black than white miners developed silicosis after relatively short periods of employment and at younger ages. The proportion of black miners with silicosis reached 2% after fewer years than it did for white miners (15–19 years versus 20–24 years), and the proportion of black miners with silicosis below the age of 50 years was almost double that of white miners (0.07 versus 0.04). These results have been reported elsewhere (1).

### Asbestos-related diseases in diamond mine workers

Of all the deceased mine workers who came to autopsy from 1975 to 2008, 1,887 had a record of having worked in the diamond-mining sector. Five hundred and fifty-nine (29.6%) had worked only in the diamond-mining sector – an exhaustive search of the multiple data sources showed no employment in any other mining sector or elsewhere – and fulfilled the study inclusion criteria (3); 24 (4.3%) had one or more asbestos-related diseases at autopsy. After a comprehensive review of all data sources to ascertain possible exposure to asbestos outside of the diamond-mining industry, only six diamond miners with one or more asbestos-related diseases were included in the case series. Four had asbestosis, one had pleural plaques, and one had malignant mesothelioma and pleural plaques.

The stored lungs of an additional 11 diamond mine workers were analysed under scanning electron microscopy (SEM). Tremolite asbestos fibres or fibres in the tremolite–actinolite amphibole asbestos series were identified in five. No chrysotile asbestos fibres were identified even though four of the five workers had worked in a diamond mine close to a chrysotile asbestos deposit. None had an asbestos-related disease.

Tailings samples from a diamond mine near a crocidolite asbestos deposit contained tremolite-actinolite series fibres. The tailings samples from the diamond mine near a chrysotile deposit contained chrysotile fibres and tremolite-actinolite series fibres. Tailings samples and dust collected from a crusher located above ground from a diamond mine near anthophyllite and chrysotile deposits contained tremolite-actinolite series fibres. A sample of pure chrysotile asbestos was also collected from between the kimberlite pipe and the country rock in this mine.

### Silicosis in platinum mine workers

A total of 12,241 men in the PATHAUT database had worked in the platinum-mining industry from 1975 to 2009. As for the diamond mine workers, a thorough search of all available data sources produced no evidence of employment in another mining sector for 6,490 (53%) of the platinum mine workers; 85 (2.2%) had silicosis at autopsy. Fibrotic nodules, a recognised precursor of silicosis, were identified in the lymph nodes of 490 (12.7%). After reviewing all available data sources, five mine workers with pulmonary silicosis (four of whom also had fibrotic nodules in the lymph nodes), plus an additional 25 with fibrotic nodules in the lymph nodes but without silicosis, were identified. There was enough evidence to suggest that these pathological changes occurred in the course of the miners’ employment in the platinum mines giving a crude autopsy prevalence of 5/6490 (0.06%) definite and 25/6490 (0.39%) potential silicosis at autopsy in platinum miners (4).

## Discussion

The high rates of silicosis are of major concern in the gold-mining industry largely because of the association of silicosis and tuberculosis (24); South African gold miners have one of the highest incidence rates of pulmonary tuberculosis in the world (25).

The alarming increase in the proportion of gold miners with silicosis at autopsy reflects the failure of the mining companies to reduce silica dust to safe levels. There is no evidence to believe that silicosis levels have decreased since the 1940s (14, 26), and this is supported by these findings. As miners continue to age and work for longer periods, the burden of silicosis and other diseases will continue to rise. This has far-reaching implications for health services that need to be prepared for increasing morbidity and mortality in current and ex-miners.

South Africa is committed to the World Health Organization/International Labour Organization initiative to eliminate silicosis by 2030. The Department of Mineral Resources (DMR) set targets to reduce 95% of all respirable crystalline silica measurements to below 0.1 mg/m^3^ by December 2008 and, after December 2013, to have no new cases of silicosis among previously unexposed individuals (27). However, the 2008 target was not met: the proportion of mines reaching 95% compliance decreased from around 94% in 2006 to less than 85% in 2010 (28). Consequently, the second milestone is also unlikely to be met. Nevertheless, silicosis rates need to be monitored by the DMR on an ongoing basis to evaluate the success of this and any other similar programmes.

The migrant labour system, together with employment practices, played a major role in the development of silicosis (29–31). Migrant labour-related problems are deep-seated within the mining industry (32), and mine occupational health professionals need to become actively engaged with management on these issues. Oscillating migration is a risk factor for work-related disease and falls within the scope of occupational health services. Oscillating migration has diluted community and organised labour pressures on mine management to reduce dust levels and control disease and has enabled mining companies to limit disease-associated costs, thereby reducing the financial incentive to control dust and disease. Policies need to be instituted to assure that ex-miners, in particular, have access to health care and compensation services. A more comprehensive discussion on this subject was published as part of the thesis (2).

The need for regional and national policies that acknowledge the economic value of migrant workers and that provide for infrastructure, such as strengthening health services where migrant workers are employed or live, and in labour-sending areas, is vital (2). As the South African mining industry shrinks, health services in labour-sending areas are becoming even more overstretched by returning migrants. Even though policies to address these and other issues might be in place, the translation into action is fraught with obstacles. This is discussed in depth in a recent paper by Murray et al. (33).

Diseases previously undocumented in diamond and platinum mine workers were found to be associated with exposure to mineral dusts and fibres. The evidence for the risk of asbestos-related diseases in diamond miners is provided by the mineralogy of kimberlite, the diagnosis of asbestos-related disease in diamond mine workers, and the identification of asbestos fibres in the lungs of diamond mine workers and diamond mine tailings. The diagnosis of silicosis and fibrotic nodules in the lymph nodes of platinum miners, as well as silica dust measurements in some of the mines, point to a risk of silicosis in platinum mine workers. Although mines are required by law to establish and maintain disease surveillance programmes (34), occupational health practitioners need to be aware that diseases other than those recognised as risks in the mining sector in which the employee works may also occur.

This work needs to be extended to identify and quantify risks in other mining sectors where occupational respiratory research has not been conducted. Many mines worldwide are ‘contaminated’ with asbestos mineral tailings (35–40). Asbestos has been reported in a vermiculite mine in South Africa (41), and there are anecdotal reports of asbestos in other mines such as the iron and manganese mines in the Northern Cape province and in chrome/platinum mine tailings dumps (James I Phillips, NIOH, personal communication). The Bushveld Complex is a mineral-rich area that is being extensively mined. The potential for exposure of mine workers in this region to associated minerals and, hence, the risk of respiratory disease, needs to be investigated in other mining sectors such as tin, vanadium, feldspar, and so forth.

The two major hindrances to this research were poorly documented work histories and lack of exposure measurements.

All mining companies should record comprehensive work histories from the time the worker is first employed including jobs outside of the mining industry. Aside from the benefits for families seeking compensation for occupational disease, this information will enable more rigorous analysis of disease associations related to different exposures during mining processes as well as during employment in different industries unrelated to mining.

Although the DMR requires all mines to conduct occupational hygiene surveillance on a regular basis, according to prescribed guidelines, many of the mines do not follow the regulations. In some platinum mines, silica levels are believed to be too low to measure, and they are commonly reported as ‘below the detectable limit’ (20). Silica dust measurements reported by gold mines are estimated, rather than measured directly, and are based on a number of extrapolations. The reliability of the data is affected by the fact that the dust measurements are collected for risk assessment in accordance with the ODMW Act for purposes of calculations of levies that the mines pay. The measurements are not validated by the DMR or any other external agency. Asbestos is also not measured routinely in most mines although there are anecdotal reports of its presence in many mine environments. Because both silica and asbestos occur throughout South Africa, it is likely that they will be present in many mines. Dust measurements should be regularly conducted by all mines and audited and validated by the DMR.

These recommendations are not restricted to South Africa. Platinum and diamond deposits are mined in Zimbabwe, Angola, the Democratic Republic of Congo, Ghana, Tanzania, Lesotho, and Botswana – in many cases by large multinationals. The recording of work histories and exposure measurements should be mandatory.

In South Africa, the quality of future research on the health of mine workers hinges on the DMR putting legislation in place regarding the documentation of comprehensive work histories and the measurement, reporting, and validation of dust levels and dust measurement procedures. Such legislation will go a long way towards protecting the health of mine workers.
